# Giant Gallbladder Revealed by Chronic Cholecystitis Gallstone: A Case Report and Review of the Literature

**DOI:** 10.7759/cureus.13906

**Published:** 2021-03-15

**Authors:** Houda Mirali, Imane Kamaoui, Tijani El Harroudi, Imane Skiker, Badr Serji

**Affiliations:** 1 Department of Radiology, Mohammed VI University Hospital, Oujda, MAR; 2 Surgical Oncology, Mohammed VI University Hospital, Regional Oncology Center, Oujda, MAR

**Keywords:** giant, gallbladder, chronic obstruction, gallstone cholecystitis

## Abstract

We report here an extremely rare case of giant gallbladder and discuss diagnostic circumstances and different ethiopathogenic theories. A 53-year-old woman presented acute right hypochondrium pain. Ultrasonography showed a huge cystic mass with gallstones and a CT scan confirmed the diagnosis of giant gallbladder. Cholecystectomy was performed. Outcomes were uneventful and histopathological examination of the specimen confirmed the presence of chronic cholecystitis.

Enlargement of the gallbladder is related to biliary retention. This enlargement is favored by the slow evolution of malignant pathologies. However, some benign situations have been reported in the literature, and giant gallbladder can occur in a benign situation even if its ethiopathogeny is not so clear.

## Introduction

Enlarged gallbladders in adults are usually observed with malignancies, especially in association with pancreatic or distal biliary tumor [1]. In benign situations, they are very rare. We report a case of giant gallbladder.

## Case presentation

**Figure 1:**
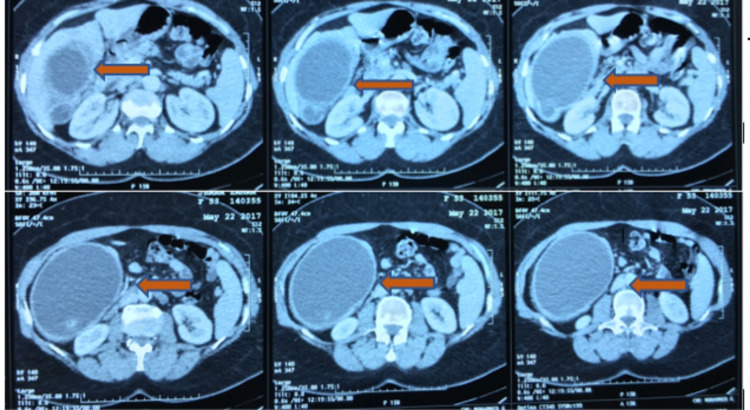
CT imaging showing a huge gallbladder with wall thickening

**Figure 2:**
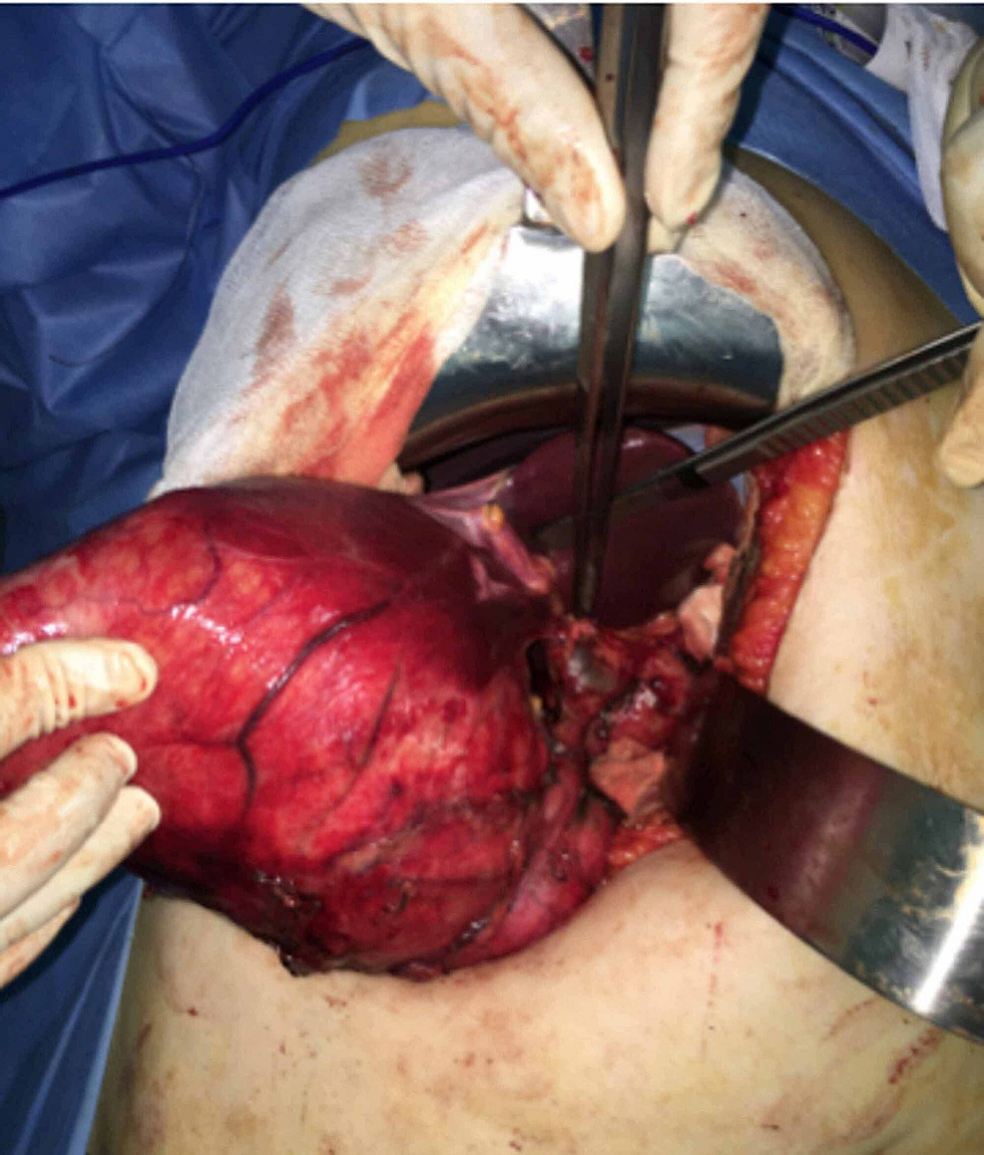
Per operative view

**Figure 3:**
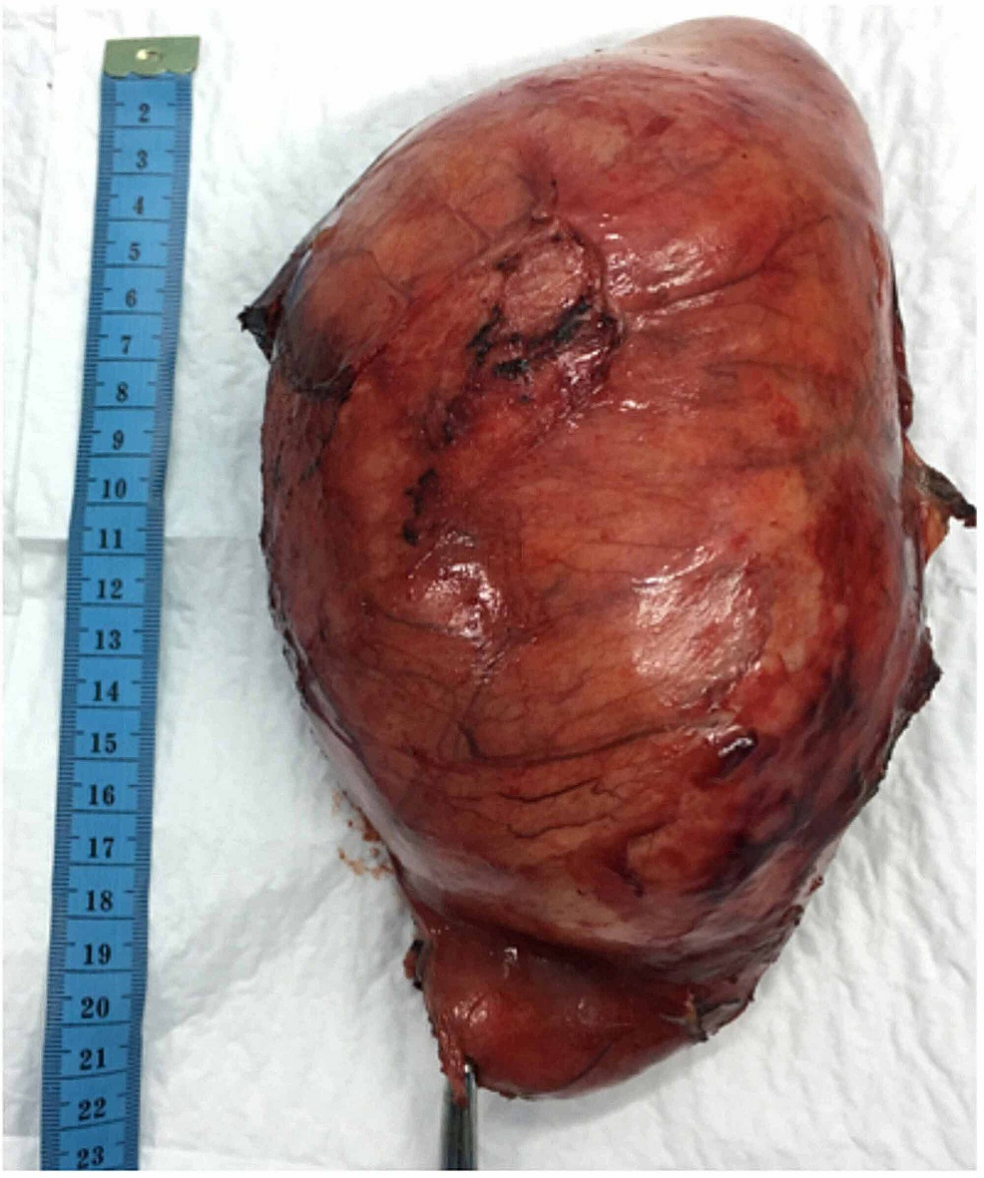
Operative part after resection

A 53-year-old woman, who had no medical history in relation to the gallbladder disease, admitted to the emergency department due to acute abdominal pain in the right hypochondrium without vomiting, fever, or jaundice. Over the past year, she had often suffered from abdominal pain, which she attributed to a colonic disorder. At admission, physical examination revealed a large, sensitive abdominal mass in the right hypochondrium that extended to the upper part of the right iliac fossa. Her body temperature was 37.1°C, pulse 81/min, and blood pressure 130/70 mmHg. Ultrasonography showed a large, well-delineated cystic mass with a wall thickness of 9 mm that contained multiple large gallstones. Imaging of the common bile duct was difficult because the intrahepatic biliary tract was not dilated, and an enlarged gallbladder was suspected. Abdominal computed tomography (CT) confirmed the diagnosis of enlarged gallbladder with massive hydrops and showed multiple gallstones with a large one at the neck of the gallbladder (Figure 1). The distal part of the common bile duct was not dilated, but the proximal part was dilated, although no intracholedochal gallstone was visible. Laboratory tests demonstrated a white blood cell count of 6600/mm3, C-reactive protein level of 15.47 mg/dL, a total bilirubin level of = 6.70 μmol/L, a gamma glutamyl transferase level of 25 IU/L, an alkaline phosphatase level of 64 UI/L, a prothrombin level of 93%, and a lipase level of 14 UI/L. The diagnosis of painful gallbladder with hydrocholecystitis was thus retained. Surgical exploration through a right subcostal incision confirmed the diagnosis. Dissection in the triangle of Callot showed that the gallbladder was exerting a mass effect on the common bile duct, which explained the proximal dilatation shown on CT (Figure 2). After the dissection of the cystic pedicle, cholecystectomy was performed. A drainage system was left for two days after surgery. The postsurgical course was uneventful, and the patient was discharged three days after the surgery. Histopathologic study confirmed the diagnosis of giant chronic cholecystitis, in which the gallbladder measured 22 x 14 x 10 cm without malignancy (Figure 3). 

## Discussion

The diagnosis of giant gallbladder may be difficult, especially in children, and can be confused with other abdominal cystic masses or with giant choledochal cyst [2,3]. In adults, enlargement of the gallbladder is usually related to biliary retention. According to Courvoisier’s law, the progressive growth of malignancy (such as a tumor in the pancreatic head or distal biliary tumor) allows the enlargement of the gallbladder over time [4]. By the same physiopathologic process, gallbladder malignancy that obstructs the cystic duct can, in very rare cases, lead to the same situation [5]. However, in very few cases reported in the literature, the presence of a hepatic disorder such as Byler’s disease or congenital large gallbladder was associated with the presence of a large gallbladder in adults without any obstructive causes [6-8]. In our patient, the enlargement of the gallbladder was associated with large gallstones. CT showed a large one localized at the neck of the gallbladder. Those stones may be mobile and may cause intermittent obstruction of the biliary cystic duct, which can lead to chronic cholecystitis, progressive enlargement of the gallbladder, and massive hydrocholecystitis. This mechanism may explain the few cases of big gallbladder with cholelithiasis reported in the literature [8-10].

Table 1 lists the few cases reported in PubMed in the English literature. The majority of cases have been in women aged > 50 years old and the reports have emphasized the length of time over which the gallbladder was enlarged.

**Table 1:** Cases of giant gallbladder reported in PubMed

Publication	Age	Sex	Diagnosis conditions	Size (mm)	Stones	Pathologist findings
Grosberg (1962) [9]	95	F	Emergency	140	Yes	Gangrenous cholecystitis
Maeda et al. (1979) [8]	36	F	Abdominal Mass	180	No	Normal gallbladder
Hsu et al. (2011) [5]	87	F	Emergency	164	Yes	Adenocarcinoma
Panaro et al. (2012) [6]	17	M	Byler’s disease	430	No	Normal gallbladder
Zong et al. (2013) [7]	55	F	Abdominal mass	300	No	Inflammatory cell infiltration
Kuznetsov et al. (2014) [10]	77	F	Emergency	240	Yes	Chronic cholecystitis
Mirali et al. (present study)	53	F	Emergency	220	Yes	Chronic cholecystitis

## Conclusions

Giant gallbladder can be one of the atypical presentations of benign biliary tract diseases. We discuss the case of a female patient with chronic hypochondrium pain. Imaging results by ultrasonography and computed tomography showed a huge gallbladder with wall thickness that contained multiple large gallstones; in addition, neither biliary duct dilatation nor tumor in the pancreatic head was demonstrated. This case report may highlight the infrequent benign situations associated with enlargement of the gallbladder.
